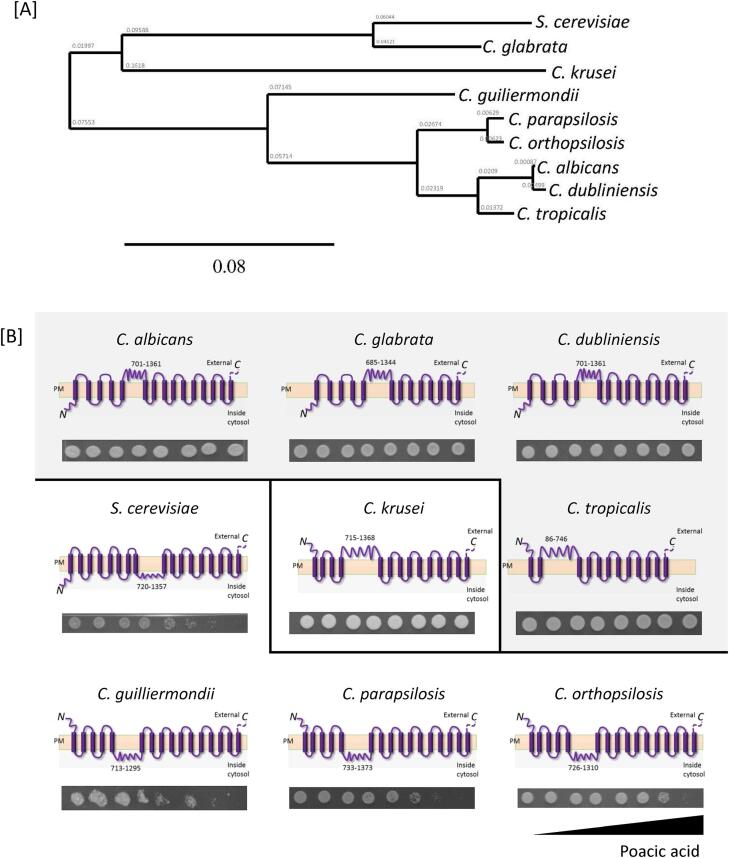# Yeast species-specific, differential inhibition of β-1,3-glucan synthesis by poacic acid and caspofungin

**DOI:** 10.1016/j.tcsw.2018.09.001

**Published:** 2018-09-26

**Authors:** Keunsook K. Lee, Karen Kubo, Jehan Abdelmoneim Abdelaziz, Iain Cunningham, Alessandra de Silva Dantas, Xiaolin Chen, Hiroki Okada, Yoshikazu Ohya, Neil A.R. Gow

**Affiliations:** a The Aberdeen Fungal Group, MRC Centre for Medical Mycology, School of Medicine, Medical Sciences & Nutrition, Institute of Medical Sciences, University of Aberdeen, Foresterhill, Aberdeen, UK; b Department of Integrated Biosciences, Graduate School of Frontier Sciences, University of Tokyo, Kashiwa, Chiba, Japan; c AIST-UTokyo Advanced Operando-Measurement Technology Open Innovation Laboratory (OPERANDO-OIL), National Institute of Advanced Industrial Science and Technology (AIST), Kashiwa, Chiba, 277-8565, Japan

**Keywords:** Antifungal agents, Glucan synthesis, Fks1, Fungal cell wall, *Candida* species

## Abstract

The rise of widespread antifungal resistance fuels the need to explore new classes of inhibitory molecules as potential novel inhibitors. Recently a plant natural product poacic acid (PA) was shown to inhibit β-1,3-glucan synthesis, and to have antifungal activity against a range of plant pathogens and against *Saccharomyces cerevisiae*. As with the echinocandins, such as caspofungin, PA targets the synthesis of cell wall β-1,3-glucan and has potential utility in the treatment of medically important fungi. However, the antifungal activity of PA against human pathogenic *Candida* species has not been explored and the precise mode of action of this compound is not understood. Here, we show that PA sensitivity is regulated by the calcineurin pathway and that susceptibility to PA varied significantly between *Candida* species, but did not correlate with *in vitro* β-glucan synthase activity, cell wall β-glucan content or the sensitivity of the species to caspofungin. Strains with point mutations (S645Y or S645P) in the hotspot1 region of the β-1,3-glucan synthase subunit Fks1, had decreased sensitivity to caspofungin but increased sensitivity to PA. *C. guilliermondii*, *C. orthopsilosis*, and *C. parapsilosis* were more sensitive to PA than *C. albicans*, *C. dubliniensis*, *C. tropicalis*, and *C. glabrata*. These observations suggest that there are significant differences in the mode of action of PA and caspofungin and that PA or PA analogues are not likely to have broad spectrum activity in the treatment of *Candida* infections.

## Highlights

• Poacic acid antifungal activity is both strains and species dependent for a range of *Candida* species.
• The calcineurin pathway regulates poacic acid sensitivity in *C. albicans*.
• Point mutations in β-1,3-glucan synthase Fks1 differentially affect poacic acid and echinocandin sensitivity.

## Graphical abstract

**Figure:**
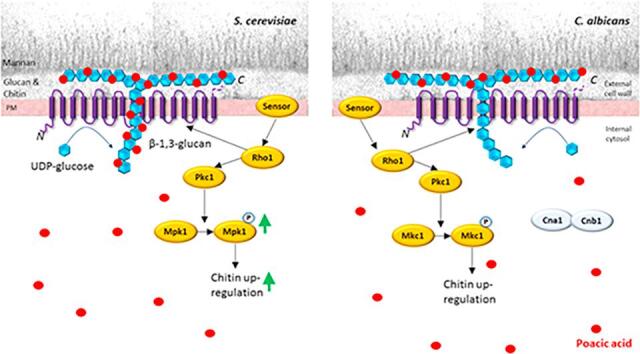

## Introduction

The fungal cell wall is a dynamic organelle that is essential for its viability. The fungal cell wall is a promising antifungal target for the therapeutic treatment of human fungal pathogens because the major components - chitin, glucan, and mannan, are absent from the human body. Depending on the fungus and growth conditions, the proportion and structural composition of cell wall components varies considerably (Free, 2013, Erwig and Gow, 2016, Gow and Netea, 2016, Gow et al., 2017). However, to date all fungi examined have β-1,3-glucan in their cell wall and this plays critical roles as a physical barrier, as a scaffold for the attachment of other cell wall components and in maintaining cell shape (Gow et al., 2017). This makes β-1,3-glucan synthesis an ideal broad-spectrum target for antifungal drugs. However, the cell wall is dynamic and can alter its structure depending on the environment and carbon source the fungi encounter, and in response to cell wall stress. Activation of the Ca^2+^/calcineurin, HOG and PKC pathways all occur in response to cell wall damage and result in the induction of compensatory mechanisms such as the synthesis of chitin (Munro et al., 2007, Munro, 2013, Gow et al., 2017). Failure to maintain cell wall integrity compromises cell viability and ultimately results in cell death (Negishi and Ohya, 2010, Rodríguez-Pena et al., 2010, Walker et al., 2013a, Walker et al., 2013b), therefore, cell wall integrity is constantly monitored via sensors located in the cell wall and membrane and at critical cell cycle check points (Roberts et al., 1983, Suzuki et al., 2004, Côte et al., 2009, Negishi and Ohya, 2010, Gow and Hube, 2012, Negishi et al., 2016).

The three echinocandins caspofungin (CSF), micafungin, and anidulafungin, are the newest class of antifungal agents on the market and have been clinically approved by the US Food and Drug Administration since 2001 (Pappas et al., 2009, Pound et al., 2010, Pfaller, 2012, Perlin, 2015b). They are non-competitive inhibitors of β-1,3-glucan synthase (Douglas et al., 1997, Odds, 2010, Nett and Andes, 2016) and therefore are not substrate analogues that bind to the enzyme active site. Indeed, the physical binding site of echinocandins to the Fks1 target protein has not been precisely defined. A number of clinical cases of echinocandin resistance have been reported (Imtiaz et al., 2012, Arendrup and Perlin, 2014, Perlin, 2015b, Sanglard, 2016) which is most commonly due to amino acid substitutions in Fks1, one of three major “hotspots” regions in the predicted external face of the β-1,3-glucan synthase transmembrane protein (Kurtz et al., 1996, Park et al., 2005, Garcia-Effron et al., 2008, Garcia-Effron et al., 2009a, Johnson et al., 2011, Johnson and Edlind, 2012, Perlin, 2015b, Kolaczkowska and Kolaczkowski, 2016, Prasad et al., 2016). For example, in *C. albicans* the serine^645^ to proline or tyrosine amino acid substitution at of Fks1 is frequently found in echinocandin-resistant strains (Perlin, 2015a, Perlin, 2015b). These mutations are often, but not always, accompanied by elevated cell wall chitin content (Ben-Ami et al., 2011, Ben-Ami and Kontoyiannis, 2012, Lee et al., 2012, Perlin, 2015a, Perlin, 2015b Perlin et al. 2015; Walker et al., 2013a, Walker et al., 2013b). However, resistance to CSF has also been described which is not associated with amino acid substitutions in Fks1. In addition, *C. albicans* strains that have an elevated chitin content in the cell wall are significantly less susceptible to CSF *in vivo* and *in vitro* (Walker et al., 2008, Lee et al., 2012, Walker et al., 2015). Paradoxical growth (also called the “Eagle effect”) of *C. albicans* at which growth still occurs at supra-MIC concentrations of the echinocandins has also been shown to be correlated with up-regulation of chitin synthesis (Stevens et al., 2006, Stevens, 2009). Furthermore, a number of CSF resistant isolates are found in other “non-*albicans*” species (Garcia-Effron et al., 2010, Perlin, 2011, Perlin, 2015b), including the emerging pathogen *Candida auris*, where as many as one third of strains are echinocandin resistant or cross-resistant (Lockhart et al., 2017a, Lockhart et al., 2017b). These health challenges highlight the need for new antifungal agents to augment the antifungal armamentarium.

Poacic acid (diferulate, 8-5-DC), PA, is a natural plant metabolite found in the lignocellulosic hydrolysates of grasses and has been characterised as a promising antifungal agent that targets β-1,3-glucan synthesis (Piotrowski et al., 2015). This compound inhibited growth of *Saccharomyces cerevisiae*, *Alternaria solani* and the oomycete *Sclerotinia sclerotiorum in vitro*. Lesion development on soybean leaves by *S. sclerotiorum* was also inhibited by PA *ex vivo*. PA apparently acts by directly binding to β-1,3-glucan polysaccharide, and therefore differs from the mode of action of echinocandins which directly target the β-1,3-glucan synthase enzyme. In this study we contrast the modes of action of two β-glucan synthesis inhibitors, poacic acid (PA) and caspofungin and show that they have distinct mode of actions and different host range activities. We determined the activity of PA against a range of clinically important fungal pathogens and demonstrate that PA is differentially active against a range of *Candida* species and examine the relationship between species-specificity, β-1,3-glucan synthesis and its sensitivity to PA. Our findings demonstrate that PA and echinocandins inhibit β-1,3-glucan synthesis in different ways and that PA-based pharmacophores may not be suitable as pan-*Candida* inhibitors.

## Results

**Fig. 1:**
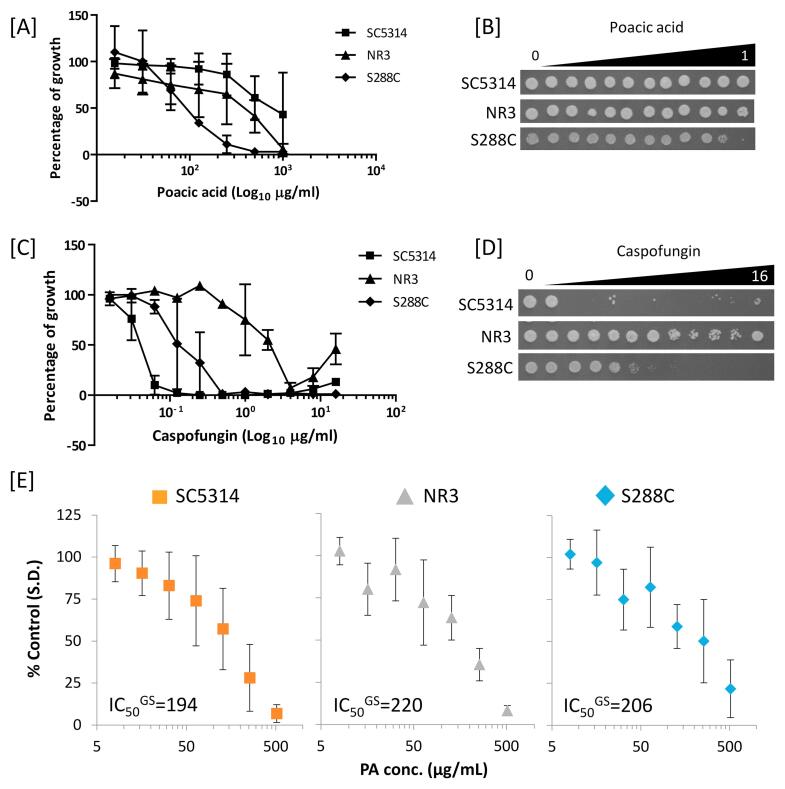
PA sensitivity and glucan synthase activity of *C. albicans.* Sensitivity of *C. albicans* SC5314 (wild type), NR3 (containing a point mutation in *FKS1*) and *S. cerevisiae* S288C to PA [A] and caspofungin [C]. Samples of 2.5 μl from the wells of the MIC plates were spotted on YPD plates and incubated at 30 °C for 24 h [B & D]. Error bars are SEM (n = 3). [E] Membranes of SC5314, NR3, and S288C were prepared from logarithmic-phase cells and glucan synthase (GS) activities were measured after solubilization of purified membrane with 0.4% Brij-35. Final concentrations of PA were 8, 16, 32, 64, 128, 256, and 512 μg/mL. All measurements were performed in triplicate. Plots of S288C, SC5314, and NR3 separately show mean values ± SD (n = 3). The half maximal inhibitory concentration (IC_50_^GS^) of S288C, SC5314 and NR3 were 206, 194 and 220 µg/ml, respectively.

Differential sensitivity of *C. albicans* and *S. cerevisiae* to PA. First we investigated the antifungal activity of PA against the *C. albicans* reference strain SC5314, and *S. cerevisiae* S288C (shown previously to be sensitive to PA) as well as the echinocandin resistant *C. albicans* NR3 strain. As shown in Fig. 1A, *S. cerevisiae* was 5- to 10-fold more sensitive (inhibitory concentration, IC_50_ = 110 µg/ml) to PA than both *C. albicans* strains, NR3 (IC_50_ = 515 µg/ml) and SC5314 (IC_50_ > 1000 µg/ml). The measured sensitivity against *S. cerevisiae* corresponds with that cited previously (Piotrowski et al., 2015). Therefore *C. albicans* SC5314 was significantly less sensitive to PA despite being echinocandin sensitive (Fig. 1C). Unexpectedly, the CSF resistant *C. albicans* NR3 strain was significantly more sensitive to PA, than SC5314. When the cells from the MIC assays were re-plated on YPD agar plates in the absence of drug the PA-treated *C. albicans* SC5314 and NR3 strains grew normally, demonstrating that PA had a fungistatic, rather than a fungicidal effect on growth (Fig. 1B and D).

Kinetic analyses revealed a correlation between the inhibition of β-1,3-glucan synthase enzyme activity and echinocandin MIC (Garcia-Effron et al., 2009b). In addition, *S. cerevisiae* β-1,3-glucan synthase activity was reduced when treated with PA and ^14^C-glucose incorporation in glucan was significantly decreased (Piotrowski et al., 2015). Therefore, we compared the effect of PA on β-glucan synthase activity using microsomal cell membrane preparations as a source of Fks1 enzyme. The inhibitory concentration (IC_50_^GS^) was determined for β-1,3-glucan synthases isolated from the total membrane fractions in SC5314, NR3, and S288C. The glucan synthase activity of all three stains was found to be inhibited to a similar extent by PA (Fig. 1E). The PA IC_50_^GS^ for β-1,3-glucan synthase of SC5314 was 194 µg/ml, which was comparable to NR3 (IC_50_^GS^ = 220 μg/ml) and S288C (IC_50_^GS^ = 206 μg/ml) suggesting that this hotspot mutation that confers echinocandin resistance is irrelevant for PA sensitivity. Therefore, in in vitro assays, PA inhibited *C. albicans* and *S. cerevisiae* β-1,3-glucan synthases to the same degree suggesting that the differential sensitivity of the *C. albicans* and *S. cerevisiae* is not due to differences in the direct action of PA on Fks1 activity.

**Fig. 2:**
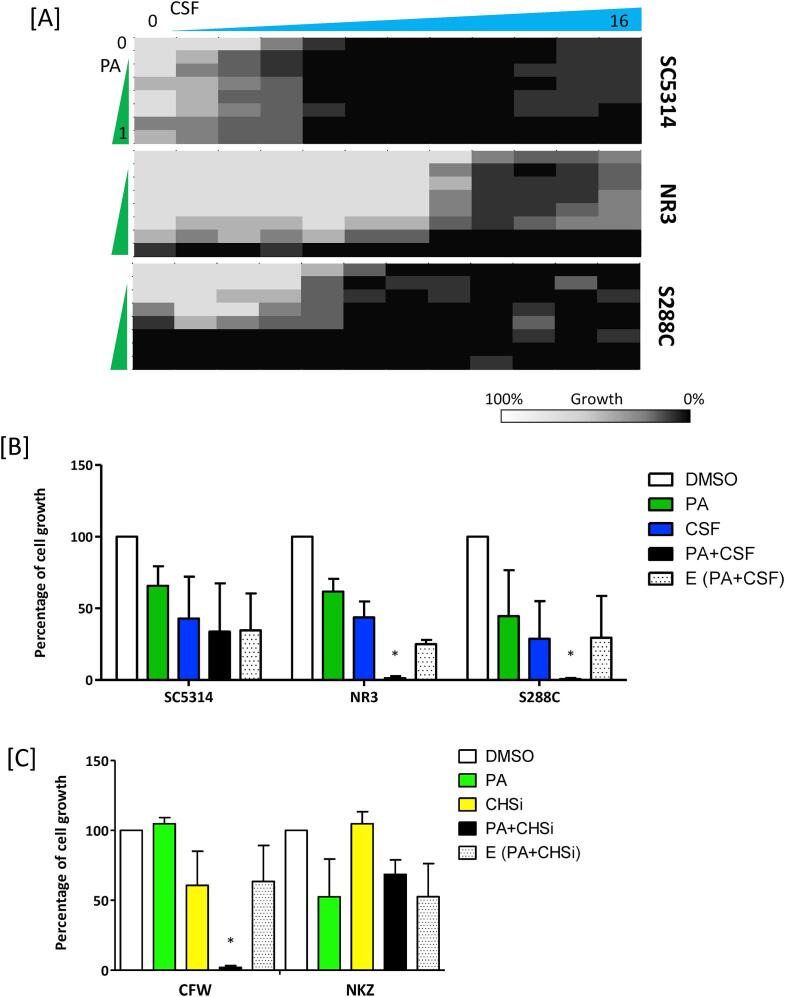
Combinatorial treatment of caspofungin and PA against *C. albicans.* [A] Sensitivity assays combining PA and caspofungin (CSF) were carried out according to CLSI methodology. Optical densities were measured after 24 h. The heat map is based on the average of three individual values expressed as percentage growth. [B] Growth inhibition due to PA in combination with CSF at sub-MIC concentration calculated as described in the Materials and Methods and presented as percentage growth compared to untreated controls. [C] Inhibition of growth in the presence of PA with or without supplementation with chitin synthesis inhibitors (CHSi) – CFW or nikkomycin Z (NKZ). *E* = expected percentage growth when both compounds are applied (See Materials and Methods). *p < 0.05 (n = 3) compared to the DMSO control.

Combinatorial synergy between PA and cell wall synthesis inhibitors. PA could potentially be useful as part of a combinatorial therapy against *Candida* infections. Piotrowski et al. demonstrated that PA and CSF acted synergistically against *S. cerevisiae* (Piotrowski et al., 2015). Therefore, we carried out sensitivity assays using combinations of PA and CSF against *C. albicans*. The results described indicate combination of PA and CSF only had a noticeable inhibitory effect the growth of both *C. albicans* and *S. cerevisiae*, compared to treatment with each inhibitor alone (Fig. 2A). Noticeably, when *C. albicans* SC5314 was treated solely with CSF, it displayed paradoxical growth at >8 μg/ml (Fig. 2A). This paradoxical growth was abolished when SC5314 was treated with both PA and CSF. Combinatorial effects calculated using the sub-MIC/IC50 values of PA and CSF, indicated mild synergist effects of additions of PA and CSF for the NR3 CSF resistant strain and *S. cerevisiae* S288C (Fig. 2B). However, combinatorial treatments of PA and two other echinocandins, micafungin and anidulafungin, did not demonstrate synergistic inhibition against the *C. albicans* wild type strain (data not shown).

Because chitin synthesis protects cells from β-glucan damage we also tested potential synergies between PA and chitin synthesis inhibitors nikkomycin Z (NKZ) (a competitive inhibitor of chitin synthase) and CFW (which can have a cidal effect against fungi by binding to nascent chitin and disrupting chitin chain maturation) (Roncero and Duran, 1985, Gaughran et al., 1994). Similar to previous findings with *S. cerevisiae* (Piotrowski et al., 2015), we observed that PA and NKZ showed no synergistic effect against *C. albicans* (Fig. 2C). However, *C. albicans* SC5314 treated with both PA and CFW exhibited a synergistic effect on growth (Fig. 2C). These results indicate that the combination of PA and CFW may enhance the cell wall polysaccharide instability, leading to enhanced killing.

**Fig. 3:**
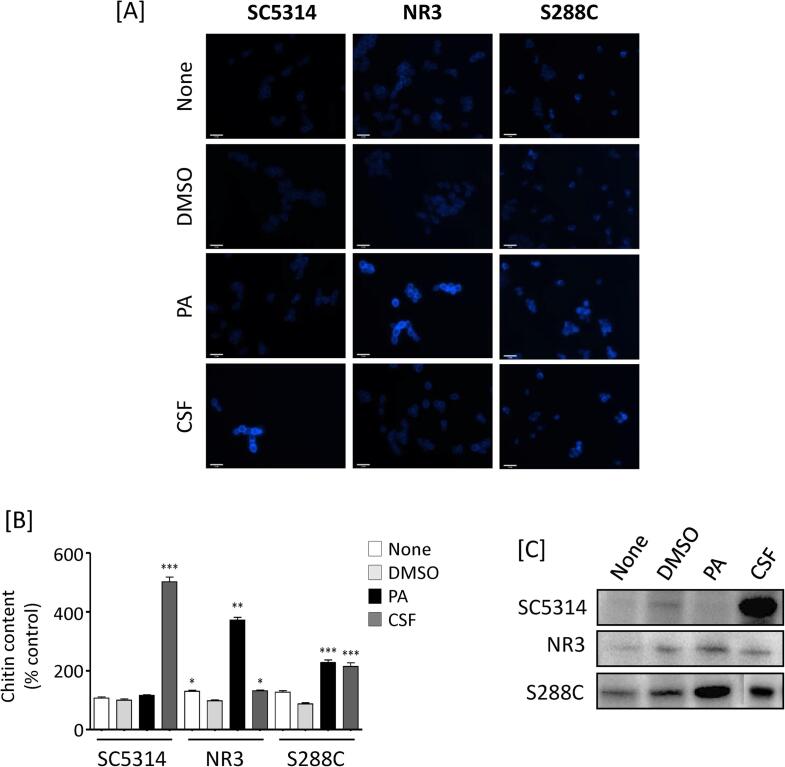
Chitin content of *C. albicans* treated with PA. [A] Cell wall chitin contents of *C. albicans* SC5314, NR3 and *S. cerevisiae* S288C treated with either PA or caspofungin (CSF). *C. albicans* and *S. cerevisiae* cells were treated with 0.5 mg/ml and 0.1 mg/ml PA, respectively and with 32 ng/ml CSF at 30 °C for 6 h. After treatment, cells were fixed and stained with 25 µg/ml CFW. Scale bars are 10 μm. [B] The fluorescent intensity was measured and expressed as percentage of control values (SC5314, no treatment, none). * *p* < 0.05. ** *p* < 0.001, *** *p* < 0.0001. Error bars = SEM (n > 50). [C] Western blot analysis of phospho-Mkc1p of *C. albicans* SC5314 and NR3, and phospho-Mpk1p in *S. cerevisiae* S288C treated with DMSO, PA, and CSF. Cells were grown at 30 °C for 4 h and treated with drugs for 10 min. Proteins were extracted and same amount of protein were loaded per lane. (None = no treatment; DMSO, 0.5% DMSO; PA, 0.5 mg/ml PA; CSF, 32 ng/ml CSF).

PA has no effect on chitin content of the *C. albicans* wild type cell wall. In fungi, β-glucan damage often leads to the induction of chitin synthesis (Munro et al., 2007, Walker et al., 2008, Lee et al., 2012). Therefore, we assessed the effect of PA on cell wall chitin measured by staining cells with CFW. DMSO treated cells of all three stains tested had no significant effect on chitin stimulation compared to the untreated controls. For wild type *C. albicans*, CSF treatments stimulated chitin synthesis, but PA did not (Fig. 3A and B). However, for the CSF resistant *C. albicans* NR3 mutant both PA and CSF stimulated chitin deposition or contents. Although microscopic observations suggested that the pattern of chitin upregulation between NR3 and *S. cerevisiae* were not identical, both PA and CSF stimulated chitin synthesis in *S. cerevisiae* (Fig. 3A and B). These data suggest that both PA and CSF compromised cell wall integrity.

Next we investigated whether the PKC/Mkc1 pathway that results in enhanced chitin formation was activated by PA (Fig. 3C). As expected, CSF treatment resulted in activation of Mkc1p/Mpk1p in both *C. albicans* and *S. cerevisiae*. Mkc1 phosphorylation was less in NR3 induced compared to SC5314 or S288C when cells were treated with CSF. PA activated Mpk1 phosphorylation in S288C, reflecting its ability to stimulate chitin synthesis in this species (Fig. 3A and B). There was no significant stimulation of Mkc1 phosphorylation in *C. albicans* SC5314 treated with PA (Fig. 3C). Therefore chitin stimulation by PA may be mediated by alternative cell wall integrity pathways.

**Fig. 4:**
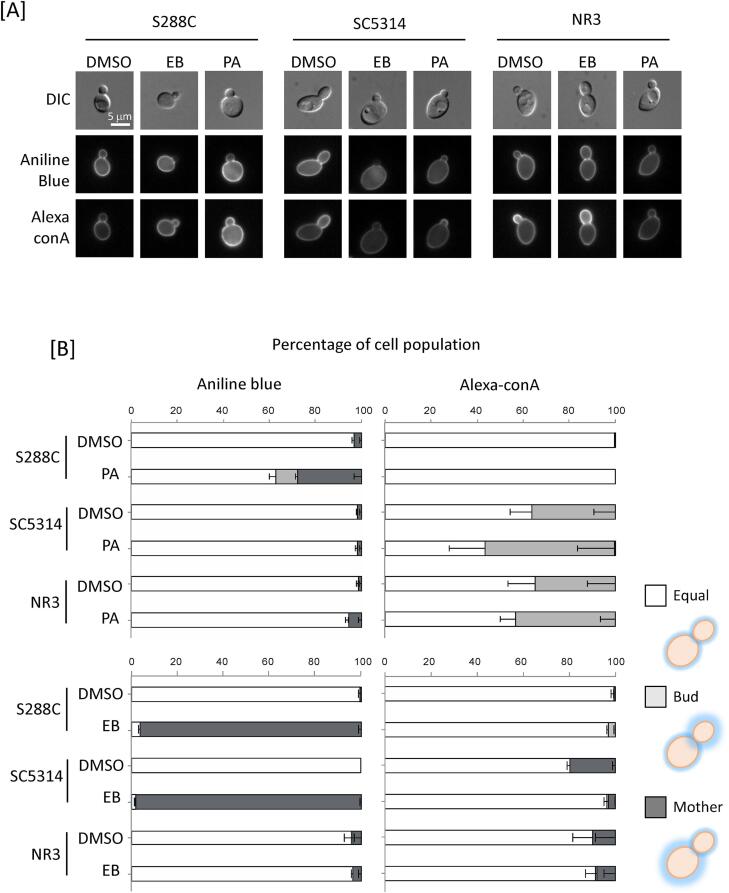

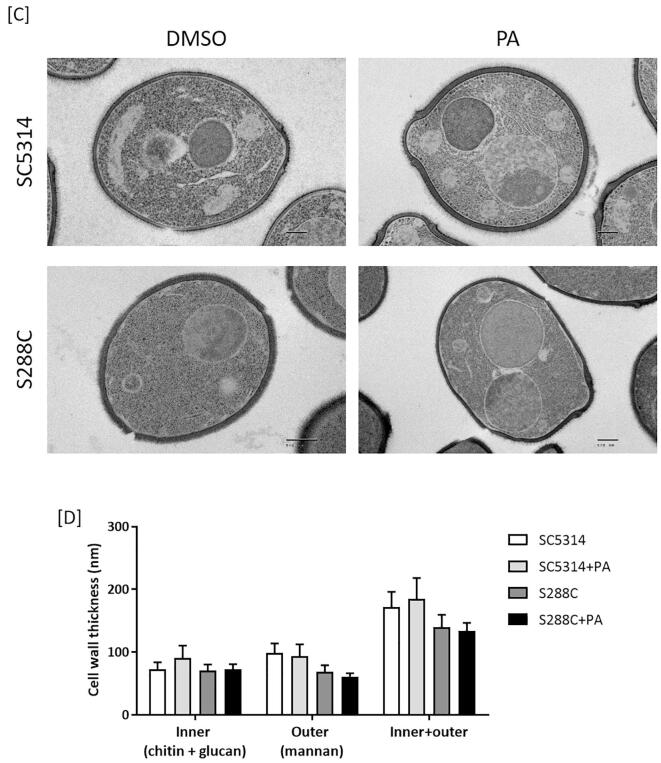
Effect on PA on mannan and glucan in *S. cerevisiae* and *C. albicans.* [A] Cells were treated with PA (PA) or echinocandin B (EB), and stained with either Aniline Blue to visualise glucan or Alexa-ConA to stain mannan. S288C and SC5314 cells were treated with 500 µg/mL PA. NR3 cells were treated with 125 µg/mL PA at 30 °C for 6 h. The three strains were treated with 4 µg/mL echinocandin B at 30 °C for 2 h. Scale bars represent 5 μm. [B] Semi-quantification of fluorescence intensity of Aniline Blue and Alexa-ConA with or without PA or echinocandin B. “Equal” represents cells where both mother and daughter cells stained uniformly. “Bud” represents cells with strong staining on daughter cells. “Mother” represents heavy staining of mother cells. Data are percentages of three independent groups. (n > 3). [C and D] *C. albicans* wild type (SC5314) and *S. cerevisiae* (S288C) cells were treated with PA 0.5 μg/ml or 0.1 μg/ml, respectively for 6 h. Scale bars = 500 nm. The thickness of the inner cell wall (chitin and glucan layer) and outer cell wall (mannoprotein fibrils only) was quantified as described in the Materials and Methods. Mean ± SD (n > 8).

PA does not affect glucan and mannan content of *C. albicans*. The impact of PA on glucan and mannan content was determined by fluorescence microscopy using Aniline Blue and Alexa-ConA, to stain β-1,3-glucan and mannan respectively. In controls, treatment of *S. cerevisiae* with PA and echinocandin B significantly decreased β-1,3-glucan staining in buds (Fig. 4A and B). Since *de novo* synthesis of β-1,3-glucan occurs at sites of budding, the reduced staining in buds implies impairment of β-1,3-glucan synthesis after drug treatment (Piotrowski et al., 2015). In contrast, PA did not alter mannan staining (Fig. 4A and B), confirming the fact that PA acts primarily on the formation of β-1,3-glucan in *S. cerevisiae* (Piotrowski et al., 2015). PA also decreased β-1,3-glucan staining in *C. albicans* buds (Fig. 4A), but this was not statistically significant at *p* > 0.05 (Fig. 4B). The echinocandin-resistant NR3 strain was unaffected by echinocandin B, but the synthesis of β-glucan of buds was slightly affected by PA (Fig. 4B). Neither PA nor echinocandin B significantly affected mannan synthesis in either *S. cerevisiae* or *C. albicans*. Reinforcing this, HFP-TEM images did not reveal any gross changes in the outer (mannan) and inner (glucan + chitin) layers of *S. cerevisiae* and *C. albicans* cell walls after PA treatment (Fig. 4C-D).

**Fig. 5:**
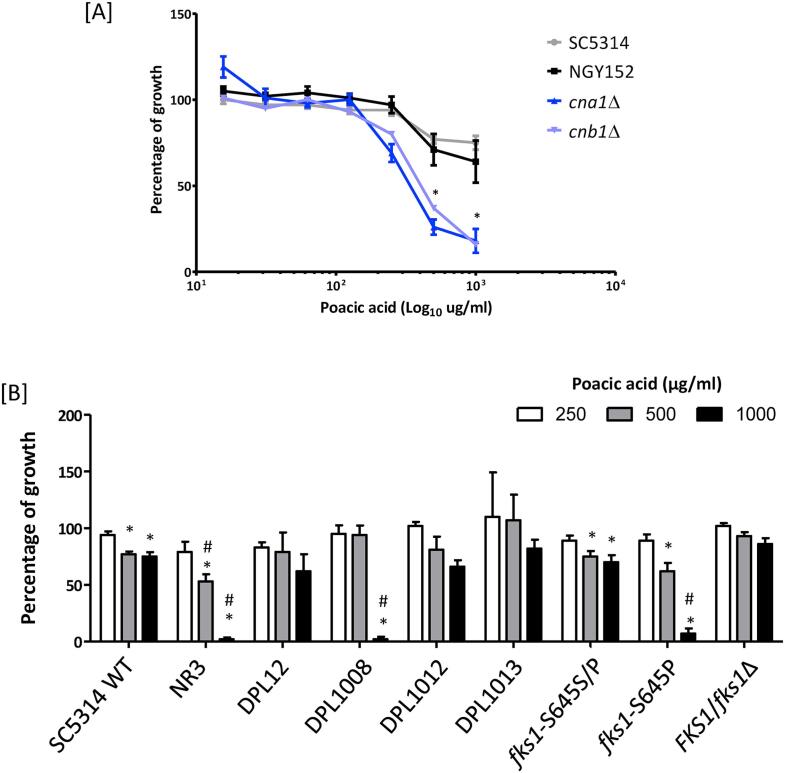
PA susceptibility of *C. albicans* cell wall associated mutants. [A] PA sensitivity of *C. albicans* mutants lacking genes encoding the calcineurin subunits (*cna*1Δ, and *cnb*1Δ) was measured relative to the control strains SC5314 and NGY152. * *p* < 0.05 compared to the controls. Error bars = SEM (n > 3). [B] MIC sensitivity of PA against to strains containing a point mutation in Fks1 or the heterozygous mutant *FKS1*/*fks*1Δ was shown as percentage of growth the DMSO control. White bars = 250 µg/ml; grey bars = 500 µg/ml; black bars = 1000 µg/ml PA. Error bars = SEM (n > 3),* *p* < 0.05 in comparison to 100% growth of each strains, #*p* < 0.05 in comparison to SC5314. 2way ANOVA test.

**Supplementary Fig. 1:**
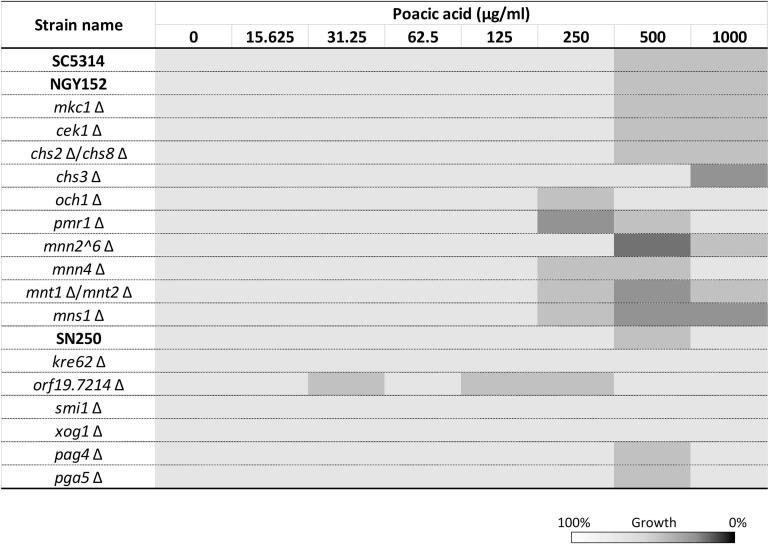


The calcineurin pathway influences sensitivity to PA. Previous studies have demonstrated that the Ca^2+^/calcineurin pathway is important for the cell remodelling and chitin upregulation of *C. albicans* in response to Ca^2+^, CFW, or/and caspofungin (Munro et al., 2007, Walker et al., 2008, Lenardon et al., 2009). To further investigate whether PA effected cell wall remodelling and integrity, a number of *C. albicans* mutants, that were defective in cell wall synthesis or cell wall integrity pathways were tested for their sensitivity to PA (the strains used are listed in Suppl. Table 1). Four groups of selected mutants were examined: 1) strains lacking genes involved in cell wall integrity pathway genes (*MKC1*, *CEK1*, *CNA1*, and *CNB1*) (Navarro-Garcia et al., 1995, Csank et al., 1998, Cruz et al., 2002, Sanglard et al., 2003), 2) mutants in cell wall chitin synthase genes (*CHS3*, *CHS2*, and *CHS8*) (Bulawa et al., 1995, Mio et al., 1996, Munro et al., 2003), 3) mannosylation mutants (*OCH1*, *PMR1*, *MNN2* family, *MNN4*, *MNT1*, *MNT2*, and *MNS1*) (Hobson et al., 2004, Bates et al., 2005, Munro et al., 2005, Bates et al., 2006, Mora-Montes et al., 2007, Hall et al., 2013), and 4) mutants in genes encoding glucan synthase, glucosidase, or glucosyltransferase (*PHR1*, *PHR2*, *CWH41*, *SMI1*, *KRE62*, C1_*14060W*, *XOG1*, *PGA4* and *PGA5*) (Fonzi, 1999, Noble et al., 2010). PA sensitivity tests were performed according to the CLSI methods (see Materials and Methods). The *phr*1Δ mutant was grown in NGY broth at pH 5 and inoculated in RPMI-1640 at pH 5 for MIC tests. Cells lacking either *CWH41* or *PHR1* showed growth defects in unstimulated conditions, and were not used for PA susceptibility testing. Only *cna*1Δ and *cnb*1Δ exhibited significantly increased sensitivity to PA (Fig. 5A). The IC_50_ values of *cna*1Δ and *cnb*1Δ were 332 μg/ml and 430 μg/ml, respectively representing a 3-fold and 2.4-fold increase in susceptibility to PA. None of the other mutants tested had significantly altered sensitivities to PA (Suppl. Fig. 1). Therefore, the calcineurin pathway is involved in PA tolerance in *C. albicans*.

Point mutations in Fks1 affecting PA sensitivity. The sensitivity to CSF can be decreased by amino acid substitutions in the Fks1 hotspot regions (Ben-Ami et al., 2011, Ben-Ami and Kontoyiannis, 2012). We tested several clinical isolates and laboratory derived strains containing point mutations in Fks1 Hotspot 1 for their sensitivity to PA (Table 1). The laboratory derived strains, *fks1*-S645S/P (a heterozygous mutant) and *fks1*-S645P (a homozygous mutation at 645 serine to proline), were generated using CRISPR-CAS9 to introduce this point mutation (see Materials and Methods). A heterozygous *FKS1*/*fks*1Δ null mutant was compared displaying marginally reduced sensitivity to PA and increased sensitivity to CSF (Fig. 5B; Table 1). A variety of point mutations in Fks1 result in elevated susceptibility to PA. Particularly strains including NR3 (S645Y), DPL1008 (S645P), and *fks1*-S645P all had significantly increased susceptibility to PA (Fig. 5B), with IC_50_s of 515 μg/ml, 767 μg/ml, and 590 μg/ml, respectively (Fig. 5B; Table 1). Amino acid substitutions in Fks1 at 641 (DPL12), 648 (DPL1012), or 689 (DPL1013) did not result in significantly altered PA sensitivity relative to the wild type control (Fig. 5B). Therefore, a variety of point mutations in Fks1 result in elevated susceptibility to PA.

**Table 1:** *C. albicans* containing an amino acid substitution in Fks1 Hotspot1.

Strain		PA IC_50_	CSF IC_50_	Fks1 Hotspot1	
	μg/ml	μg/ml	Position	Sequence	
Reference	SC5314	>1000	0.032	WT	FLTLSLRDP
Clinical strains	DPL12	>1000	0.545	F641S	**S**LTLSLRDP
	DPL1008	**767**	**3.500**	**S645P**	FLTL**P**LRDP
	DPL1012	>1000	0.340	D648Y	FLTLSLR**Y**P
	DPL1013	>1000	0.600	P649H	FLTLSLRD**H**
Lab strains	NR3	**515**	**2.300**	**S645Y**	FLTL**Y**LRDP
	*fks1* S634	>1000	1.300	S645S/P	FLTL(S/**P**)LRDP
	*fks1* S645P	**590**	**3.700**	**S645P**	FLTL**P**LRDP
	*FKS1*/*fks*1Δ	>1000	0.016	WT/Δ	FLTLSLRDP

**Fig. 6:**
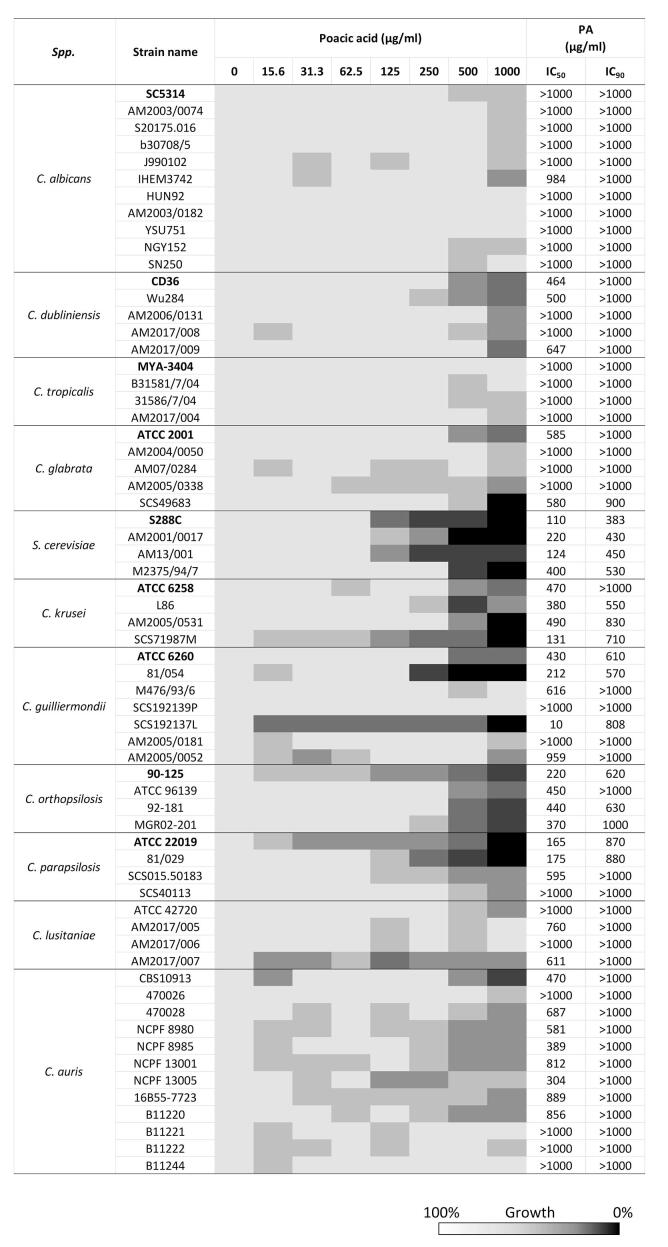
PA susceptibility of *Candida* species. Ten *Candida* species plus *S. cerevisiae* were tested for their relative PA susceptibility. Strains in bold are the reference strain for each species. The inhibitory concentrations (IC) at 50% and 10% growth were determined by analysing a locally weighted scatterplot smoothing (LOWESS) method.

Sensitivity of PA against various *Candida* species. We next investigated PA susceptibility against 10 different *Candida* species (63 strains) and four strains of *S. cerevisiae* (S288C, AM2001/0017, M2375/94/7, and AM13/001) (Fig. 6; Suppl. Table 1). The *S. cerevisiae* strains were most sensitive to PA. *C. krusei*, *C. parapsilosis*, and *C. orthopsilosis* were more sensitive to PA compared to *C. albicans* (Fig. 6). Compared to those *Candida* species, *C. guilliermondii* showed the most variable PA sensitivity. For example, the *C. guilliermondii* strains ATCC 6260, 81/054, and SCS192139L were relatively susceptible to PA (IC_50_ = 10 to 430 μg/ml), whereas M476/93/6, SCS192139P, AM2005/0181, and AM2005/0052 were somewhat less sensitive to PA (IC_50_ = 616 to > 1000 μg/ml). This species is also variable in its sensitivity to CSF (Walker et al., 2013a, Walker et al., 2013b). Interestingly, *C. glabrata* was relatively less sensitive to PA compared to *S. cerevisiae* despite of phylogenetical similarity between two species. Furthermore, *C. albicans*, *C. dubliniensis*, and *C. tropicalis* were relatively less sensitive to PA compared to *S. cerevisiae*. Of the more uncommon *Candida* species, *C. lusitaniae* and *C. auris* were generally less sensitive to PA. Therefore, PA sensitivity was both species- and strain-dependent.

**Fig. 7:**
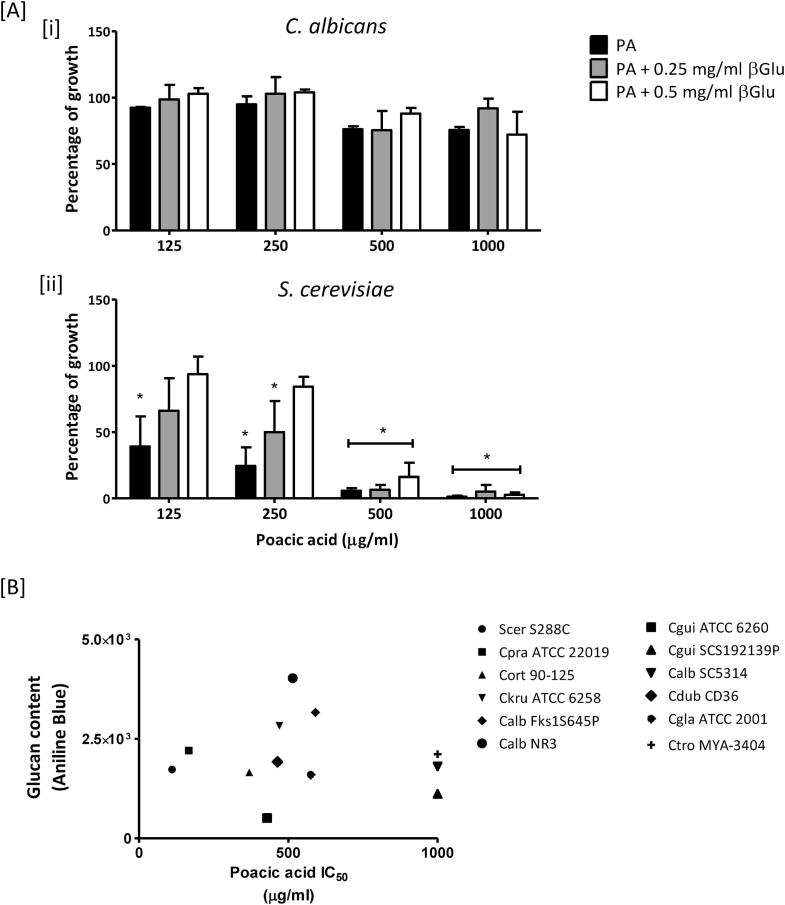
Cell wall glucan contents in different *Candida* species and *S. cerevisiae.* [A and B] MIC of PA was calculated with exogenous β-1,3-glucan (βGlu) at 0.25 mg/ml or 0.5 mg/ml. *C. albicans* wild type [i] and *S. cerevisiae* [ii] were treated with PA only or PA with βGlu. Results are expressed as percentage of growth relative to the control (DMSO). Error bars = SEM (n > 3). * *p* < 0.05. 2way ANOVA test. [B] Correlation between glucan content and PA IC_50_ values of different *Candida* species. Cells were grown exponentially for 6 h in YPD and washed with PBS. Cells were then fixed with 4% formaldehyde. Aniline Blue (25 µg/ml) was used to stain cell wall β-glucan. Fluorescence levels of individual cells (1 × 10^4^) were measured by FACS. The IC values were calculated using LOWESS analysis based on the sensitivity assay.

β-glucan content does not correlate with the sensitivity of PA to different *Candida* species. PA has been reported to bind directly to β-1,3-glucan (Piotrowski et al., 2015). We therefore investigated whether exogenous β-1,3-glucan could influence PA sensitivity (Fig. 7A). As a control, *C. albicans* and *S. cerevisiae* were grown in the medium only with exogenous β-1,3-glucan at 0.5 mg/ml. Under these growth conditions, the presence of exogenous β-1,3-glucan (0.5 mg/ml) did not impact cell growth of either *C. albicans* and *S. cerevisiae* (data not shown). In *C. albicans* the PA sensitivity was hardly affected by exogenous β-1,3-glucan at 0.25 mg/ml and 0.5 mg/ml (Fig. 7Ai). On the other hand, the growth of *S. cerevisiae* was ameliorated by addition of exogenous β-1,3-glucan in a dose-dependent manner (Fig. 7Aii). Therefore, the sensitivity of *S. cerevisiae* to PA was influenced by exogenous β-1,3-glucan in a PA-concentration-dependent manner (Fig. 7Aii).

Next we hypothesized that varied basal levels of glucan in different *Candida* species may influence PA sensitivity. To test this, the β-1,3-glucan content in *Candida* cells was quantified using Aniline Blue staining by FACS (Fig. 7B). *C. albicans* NR3 had the highest glucan content and *C. guilliermondii* ATCC 6260 had the lowest among the strains tested, and the corresponding IC_50_ values of these strains were 515 μg/ml and 430 μg/ml. Additionally, the glucan contents of *C. albicans* wild type (SC5314) and *S. cerevisiae* S288C were comparable, but the IC_50_ values were 111 μg/ml and > 1000 μg/ml, respectively. Therefore, the PA sensitivity did not correlate simply with the basal β-1,3-glucan content of the cell wall of different yeast species.

**Supplementary Fig. 2:**
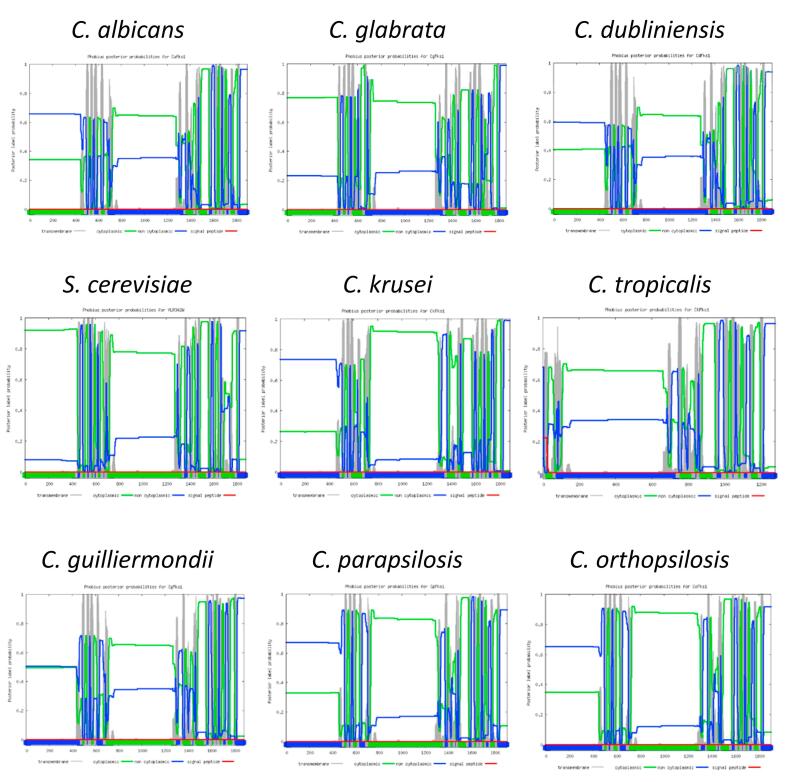


Correlation between computational prediction of β-1,3-glucan topology and PA sensitivity. The predicted transmembrane domains of *C. albicans* Fks1 were highly conserved in *C. dubliniensis* and *C. tropicalis* Fks1 but different in *S. cerevisiae* Fks1 and *C. glabrata* Fks1 (Suppl. Figs. 2 and 3). We noted a significant correlation between PA sensitivity and Fks1 active domain topology of five species (*C. albicans*, *C. dubliniensis*, *C. tropicalis*, *C. glabrata*, *C. krusei*, and *S. cerevisiae* (p < 0.05 according to Fisher’s exact test; Suppl. Table 3). One Fks1 domain was predicted to be located on the cytosolic face of the membrane for the most PA sensitive species - *C. guilliermondii*, *C. parapsilosis*, *C. orthopsilosis*, and *S. cerevisiae*) (Suppl. Fig. 3). However, the topology and function of this domain has yet to be examined experimentally.

## Discussion

The natural plant metabolite PA has been shown to have antifungal and anti-oomycete activity for a range of plant pathogens. Here we show that PA has antifungal activity against some, but not all, of major species of the human fungal pathogens from the genus *Candida*. Despite of the demonstrated potency of PA against *S. cerevisiae* (Piotrowski et al., 2015), our results demonstrated that PA did not have pan-*Candida* activity, and is therefore unlikely to be useful as a pan-broad spectrum antifungal agent. Although the mode of action of PA is not understood in detail our results support the hypothesis that β-1,3-glucan binding is important for PA action. This was underlined by observations that exogenous β-1,3-glucan attenuated PA inhibition – Most likely by removing PA from solution. β-glucan synthesis involves synthesis of β-1,3-glucan oligomers (nascent short polymers) by the Rho1-dependent β-1,3-glucan synthases Fks1, Fks2 and Gsc1 using UDP-glucose as the substrate (Douglas et al., 2001). The nascent polysaccharide matures into a β-glucan triple helix matures and subsequently undergoes structural modifications and crosslinking via a number of transglycosidase and glucan modifying enzymes such as Phr1, Phr2, the Chr family, Pga4, Pga5, Xog1, Eng1, Bgl2, and Kre62 (González et al., 1997, Plaine et al., 2008, Arroyo et al., 2016, Aimanianda et al., 2017). Our results showed that the β-1,3-glucan synthase activity in *in vitro* membrane preparations was inhibited to a similar extent by PA in both *C. albicans* and *S. cerevisiae* (Fig. 1), suggesting that effect of PA may not influence Fks1 catalytic activity directly and that differences in sensitivity to PA may be dependent on access of this plant metabolite to β-1,3-glucan in the cytosol. Furthermore, mutants lacking *XOG1*, *SMI1*, *KRE62*, *PGA4* or *PAG5*, which are all involved in β-1,3-glucan synthesis, were not altered in PA sensitivity (Suppl. Fig. 1). NMR analysis has demonstrated that the β-glucan of *C. albicans* hyphal cell walls is a cyclic glucan different from the linear glucans found in yeast cells (Kruppa et al., 2011, Lowman et al., 2014). Although it is unclear where the exact binding site of PA, it is possible that structure of β-1,3-glucan is important for the ability of nascent β-1,3-glucan to mature and that PA binding to this polysaccharide compromises its structural integrity in an analogous way to Calcofluor White binds to, and destabilises, β-1,4-linked *N*-acetylglucosamine of chitin. However, because PA binds directly to β-glucan rather than the Fks1 protein, it is possible that the Fks domain we have highlighted that correlates with PA sensitivity is involved in β-glucan binding – The product of Fks1 activity. In this model, for PA to efficiently inhibit β-glucan synthesis, binding to this polysaccharide may occur immediately after the nascent polysaccharide is formed. This binding may in turn compromise β-glucan fibril maturation and ultimately its physical strength.

We showed that point mutations in *Candida* Fks1 impacted differentially on PA and CSF susceptibility and that the transmembrane topology that was determined *in silico* the position of the predicted catalytic domain of Fks1 correlated with PA sensitivity. In *S. cerevisiae*, the Fks1 protein is predicted to have 18 transmembrane helixes (TMHs) (Johnson and Edlind, 2012). The central domain (residues ∼715–1300) is highly conserved and required for enzyme function, leading to the hypothesis that it may be associated with UDP-glucose binding and catalysis (Johnson and Edlind, 2012). Computational predictions of topographical models of this domain suggested that its localisation in the plasma membrane may be species-dependent and that it may have either an intracellular or extracellular localisation. Our data, and that published previously, suggest that PA binds β-1,3 glucan directly and that this event cause the polysaccharide instability leading to cell wall damage. High PA sensitivity correlated with a theoretical predicted intracellular location of this central domain – A hypothesis that remains to be tested experimentally. We hypothesize that if PA binding is intracellular it may immediately bind nascent β-glucan leading to more profound perturbation of the structural properties of this structural polysaccharide. Therefore, PA activity may be based on its ability to directly interact with β-glucan rather than its direct effect on β-1,3-glucan synthesis. However, the marked difference in the sensitivity of medically important species to PA suggests that it may have limited application as a lead compound informing new therapeutic strategies in medical mycology.

The structural integrity of the cell wall is monitored by membrane sensors that feed into the PKC/Mkc1 pathway and Hog1 and Ca^2+^/calcineurin pathways (Munro et al., 2007). In *C. albicans*, PA did not activate the PKC/Mkc1 cell wall salvage pathway that would be normally associated with upregulation of the chitin content and cell wall remodelling in the cell wall (Fig. 3, Fig. 4). Deletion of *MKC1* in *C. albicans* did not impact on the PA sensitivity compared to the wild type control (Suppl. Fig. 1), although other genes involved in the PKC/Mkc1 pathway were not tested. In a previous study (Piotrowski et al., 2015), *S. cerevisiae* mutants including *rom*2Δ, *pkc*1Δ, and *bck*1Δ involved in the PKC pathway showed significantly increased sensitivity to PA. Deletion of *NBP2* (encoding the Hog1-negative regulator) and *PTS1* (encoding a protein phosphatase) resulted in increased resistance to PA. Furthermore, *S. cerevisiae* Sur1 and Csg2, which are involved in mannosylinositol phosphorylceramide synthesis, activate the PKC pathway and are resistant to PA when the cognate genes are deleted. It is however possible that PA treatment could stimulate cell wall remodelling by via PKC/Mkc1 independent mechanisms (Santiago et al., 2010).

The Ca^2+^/calcineurin pathway is also important for cell wall remodelling and stress responses to caspofungin (Bader et al., 2006, Munro et al., 2007, Walker et al., 2008, Singh et al., 2009, Kaneko et al., 2010, Lafayette et al., 2010). We showed here that deletion of *CNA1* or *CNB1* increased the CSF sensitivity in *C. albicans*. The chitin up-regulation response to caspofungin in *C. albicans* is also calcineurin-dependent (Walker et al., 2008). Previously a screen of *S. cerevisiae* mutants did not reveal a role for the Ca^2+^/calcineurin pathway in determining PA sensitivity (Piotrowski et al., 2015). In contrast we showed in *C. albicans* that *cna*1Δ and *cnb*1Δ were hypersensitive to PA (Fig. 5A).

The accumulation of point mutations in one of three hot spot regions of Fks1 has been shown to confer echinocandin resistance (Garcia-Effron et al., 2009a, Garcia-Effron et al., 2010, Perlin, 2011, Arendrup and Perlin, 2014, Perlin, 2015b, Kolaczkowska and Kolaczkowski, 2016). In addition, inherent caspofungin or micafungin sensitivity is known to be variable among *Candida* species independent of the presence of Fks1 point mutations (Walker et al., 2013a, Walker et al., 2013b, Arendrup and Perlin, 2014, Perlin et al., 2015, Wanjare et al., 2016). We demonstrate that, PA sensitivity also differed between *Candida* strains. Cell wall chitin can compensate for β-glucan damage (Walker et al., 2008, Ben-Ami et al., 2011), but again there was no observed correlation between chitin content and PA sensitivity. In addition, our data demonstrated that β-1,3-glucan contents of *Candida* species varied significantly (Fig. 7), however, PA sensitivity did not correlate with gross glucan content of different *Candida* species.

In summary, our data suggest that PA and echinocandins inhibit β-1,3-glucan synthesis by different mechanisms and that the extent of PA inhibition is fungal species-specific. It is likely that PA does not directly interfere with the catalytic process of forming β-1,3-glucan at the cytoplasmic face of the membrane, but rather interferes with β-1,3-glucan maturation leading to a weaker cell wall. The variability in the ability of PA to inhibit a range of *Candida* pathogens limits its potential for development as a novel antifungal agent.

## Materials and Methods

### Strains, media, and chemicals

The fungal strains used in this study are listed in Suppl. Table 1. All strains of *Candida* species and *S. cerevisiae* were grown on an YPD agar plate (1% yeast extract, 2% peptone, 2% glucose, and 2% agar). For overnight cultures, single colonies from the agar plates were inoculated in either YPD or NGY broth (0.1% yeast extract, 0.1% neopeptone, and 0.4% glucose), and incubated at 30 °C with shaking at 200 rpm. Uridine (25 μg/ml) was supplemented in the medium for uridine auxotrophic mutants. CSF was obtained from the Aberdeen Royal Infirmary. Echinocandin B was generous gift from Osamu Kondo (Chugai Pharmaceutical Co., Tokyo, Japan).

### Antifungal susceptibility test

The antifungal susceptibility of PA against various *Candida* species and *S. cerevisiae* strains was determined according to CLSI M27-A3 guidelines in using modified RPMI-1640 (2 mM L-glutamine, 0.2 M MOPS, 2% glucose (w/v), pH7.2) as described previously (Lee et al., 2012). Briefly, fungal cells were grown in NGY at 30 °C with shaking at 200 rpm overnight to reach a stationary phase. Overnight cultures were washed with distilled water and then inoculated in RPMI-1640 (with/without drugs) to reach approximately up to 5 × 10^4^ cells/ml, and incubated at 37 °C for 24 h in a static incubator. PA concentrations ranged from 0 to 1000 μg/ml, and CSF concentrations from 0 to 16 µg/ml. The glucan extracted from *Euglena gracilis* (Sigma, 89862-1G-F) was dissolved/gelatinised in DMSO, and used in some experiments in which β-glucan was provided exogenously. After 24 h incubation in microtitre plates, the optical density of wells was measured using a VERSAmax plate reader (Molecular Devices, USA) at 405 nm. For the post-sensitivity assays of cell viability, 2.5 μl samples were taken from the sensitivity assay microtitre plates and were spotted on a fresh YPD and incubated at 30 °C overnight. The inhibitory concentration of MIC plates at 50% cell survival (IC_50_) or a 10% cell survival (IC_90_) was calculated from LOWESS curves using GraphPad Prims 5 (v5.04).

### Combinatorial sensitivity assays

Combinatorial inhibition assays were performed to investigate synergistic effect of PA with echinocandins. Growth conditions were identical to that used in antifungal susceptibility tests described above. A 12 × 8- or 8 × 8-dose-defined matrix was used in 96-well plates containing PA (0.98 to 1000 μg/ml) in combination with either CSF (0.032–16 μg/ml), NKZ (0.625–40 μM), or CFW (0.2–200 μg/ml). After incubating for 24 h at 37 °C, the final OD at 405 nm was determined. Results are presented as a heatmaps or a bar charts of the percentage of growth inhibition observed. Synergistic effects were calculated as described (Piotrowski et al., 2015) using the formula *E* = A × B/C (where *E* is the expected percentage of inhibited growth; *A* is the percentage growth of when compound A (PA) is applied alone; *B* is the percentage growth when compound B (CSF) is applied alone; *C* is the percentage growth DMSO control. Sub-MIC or IC_50_ concentrations of PA or caspofungin were used for analysis of synergistic effects. Combinations of 1000, 500 and 62.5 µg/ml PA and 0.062, 2, and 0.25 µg/ml CSF for SC5314, NR3 and S288C respectively were used. A combination of 500 µg/ml PA and 25 µg/ml CFW or 40 µM NKZ were used for SC5314.

### Semi-quantification of cell wall chitin

Cells treated with either PA or CSF were fixed with 10% neutral buffered formalin and stained with 25 µg/ml CFW to visualise cell wall chitin. Samples were observed by differential interference contrast (DIC) microscopy as described in the previous study (Walker et al., 2008, Lee et al., 2012) using a Zeiss Axioplan 2 microscope to observe samples. The Openlab software (Openlab v 5.02) was used to take images of samples and the CFW fluorescent intensity of individual cells was measured using ImageJ (v1.47).

### Mannoprotein and glucan staining

β-1,3-glucan was stained with Aniline Blue (016-21302; Wako Chemicals). Mannoprotein was stained with Alexa594-ConA (C11253; Life Technologies). β-1,3-glucan and mannoprotein were stained as described previously (Piotrowski et al., 2015, Okada et al., 2014, Okada and Ohya, 2016) with slight modification. Briefly, over-night cultured yeast cells were cultured in YPD with PA (500 μg/ml for S288C and SC5314 or 125 μg/ml for NR3) or with echinocandin B (4 μg/ml) at 30 °C. Then, cells were collected at 2 h (for echinocandin B) or 6 h (for PA) after treatment and stained with Alexa594-ConA and Aniline Blue without fixation. Cells mounted on a glass slide were exposed to UV for 30 s to bleach out PA fluorescence before acquiring images.

For fluorescence-activated cell sorting, applied in flow cytometry (FACS) analysis, cells were first grown in YPD at 30 °C for 6 h. Cells were washed twice with PBS, and fixed in 3.7% formaldehyde. Fixed cells were diluted in PBS to reach 1 × 10^6^ cells/ml, 0.5 mM EDTA was added, and samples were sonicated in a water bath for up to 10 min to disrupt cell clumps. Aniline Blue (25 μg/ml) was added immediately before FACS analysis. Unstained controls were used to establish the basal level of fluorescence. A total of 10,000 cells per sample were analysed by FACS according to BD Fontesa manufacturer’s guidelines (BD Biosciences). Data obtained by the BD FACSDiva software were transferred and analysed by FlowJo (v10.02) to semi-quantify the fluorescence intensity representing β-glucan contents.

### *In vitro* β-1,3-glucan synthase activity

To prepare membrane microsomal fractions, cells were grown at 30 °C to a density of 2–4 × 10^7^ cells/ml. The following procedures were carried out at 4 °C. Cells were harvested, washed with 1 mM EDTA, and disrupted by vortexing 4 times for 2 min each with 5 ml of glass beads in 10 ml of breaking solution containing 0.5 M NaCl, 1 mM EDTA with 1 mM phenylmethylsulfonyl fluoride. After centrifugation at 1,200 × g for 5 min, the supernatant was collected and transferred to 33 PC tubes (Hitachi, Japan). Pellets were resuspended in breaking solution, and centrifuged at 1,200 × g for 1 min. The supernatant was also transferred to new 33 PC tubes. Membrane fractions in 33 PC tubes were collected by centrifugation at 100,000×*g* for 30 min in an RP70T-203 rotor (Hitachi) with Himac CP 65β (Hitachi). The resultant microsomal membrane pellet was suspended in membrane buffer containing 50 mM Tris-HCl, pH 7.5, 10 mM EDTA, 1 mM β-mercaptoethanol, and 33% glycerol, homogenized, and stored at − 80 °C to obtain membrane mixture.

β-1,3-glucan synthase activity was measured as described previously (Piotrowski et al., 2015) with slight modifications. First, a reaction mixture was prepared containing 25 mM Tris·HCl pH 7.5, 25 mM potassium fluoride, 0.5 mM EDTA, 0.4% Brij-35, and 0.2 mM UDP-Glc (with 57 Bq UDP-[Glucose-^14^C]; NEC403; PerkinElmer). Brij-35 was used for membrane solubilisation was included in the reaction solutions for assays of *C. albicans* β-1,3-glucan synthase activity (Frost et al., 1994). Membrane fractions containing 50 μg total protein was included in the membrane mixture containing 2.0 μM GTP-γ-S and different concentrations of PA. The membrane fractions (20 μl) were added to 80 μl of the reaction mixture, incubated at 30 °C for 30 min, and stopped by the addition of ethanol. Trapping β-1,3-glucan polymer from the sample was collected by membrane filtration (cellulose ester membranes; 0.2 μm in pore size; ADVANTEC), and washed one time with 1 ml distilled water. Membrane filters were dried at room temperature for 2 h, and measured the radioactivity by a scintillation counter (LSC-6100; Aloka) with 2.5 ml scintillation mixture (Econofluor-2; PerkinElmer). The half maximal inhibitory concentration (IC50^GS^) was calculated after fitting with Gamlss beta in the R Gamlss package.

### High Pressure, freeze substitution transmission electron microscopy (HPF-TEM)

HPF-TEM was performed as described previously (Hall et al., 2013) with some modification. *C. albicans* SC5314 and *S. cerevisiae* S288C were grown in YPD at 30 °C overnight. The following day, overnight cultures were diluted in a fresh YPD medium to an OD_600_ of 0.2 and incubated at 30 °C for 1 h. Then cells were treated for further 6 h with DMSO (0.5% for SC5314 or 0.1% for S288C) or PA (500 μg/ml for SC5314 or 100 μg/ml for S288C). After incubation, cells were collected and washed with mQ-water. Cells were freeze-substituted by using a Leica EM AFS2 automatic freeze substitution system (Leica Micro-systems). Further steps were carried out according to the previous study (Hall et al., 2013). To quantify the length of the inner (chitin + glucan) and outer (mannan fibrils) layers, images were transported into ImageJ (v1.47), and 5–12 cells were randomly selected, and 5–45 measurements made from the different angles of each cell. The results present as a mean (nm) with SEM.

### Mkc1 phosphorylation analysis by western analysis

Western analysis was performed using methods described previously with modifications (Munro et al., 2007). Overnight cultures of *C. albicans* wild type, *C. albicans* NR3 (Fks1-S645Y), and *S. cerevisiae* were diluted in fresh YPD and then incubated for 4 h at 30 °C with shaking until they reached mid-log phase. Cells were then treated for 10 min with appropriate treatments, with equivalent non-treated cells used as a control. After treatments, cells were collected, and their total protein complement was extracted.

Proteins (15 µg) were separated by SDS–PAGE (polyacrylamide gel) electrophoresis; NuPAGE®Novex Bis-Tris 4–12% Precast gels (Invitrogen). Blotting was performed according to the manufacturer’s instructions. A mix of the SeeBlue® Plus2 Pre-stained (Invitrogen) and MagicMarkTM standards (Invitrogen) were loaded as markers. After blotting, separated proteins were transferred onto PVDF membranes which were rinsed with PBS and blocked in PBS-T 10% BSA (0.1% Tween-20, 10% BSA in PBS) for 30 min at RT. Membranes were then incubated overnight at 4 °C with Phospho-p44/42 MAP Kinase (Thr202/Tyr204) Antibody (Cell Signalling Technology) in PBS-T 5% BSA (0.1% Tween-20, 5% BSA in PBS). For secondary antibody staining, the membranes were incubated at RT for 60 min in PBS-T 5% BSA containing an anti-rabbit IgG, HRP-linked Antibody (Cell Signalling Technology). Signals were enhanced by SuperSignal® West Femto Maximun Sensitivity Substrate (Thermo Scientific) according to the manufacturer’s instructions. Chemiluminescence signal detection was performed using the Fusion image acquisition system v15.15 (PeQLab Biotechnologie GmbH, Germany).

### Creating a mutant containing Fks1 amino acid substitution

Amino acid substitutions in Fks1 (orf19.2929/C1_02420C) were introduced in *C. albicans*, using the CRISPR-Cas9 system according to a previous study with modifications (Vyas et al., 2015). A single guide RNA was generated using primers of Fks1sgRNA-Fwd and Fks1sgRNA-Rev (Suppl. Table 2). The single guide RNA targeting the *FKS1* locus was ligated into the plasmid, pV1200. Positive clones were selected on LB plate containing 100 μg/ml ampicillin and 50 μg/ml nourseothricin. Constructs were confirmed by sequencing. Before, transformation, the plasmid pV1200 + *FKS1*sgRNA (2–3 µg) for the Cas9 system was digested with *Kpn*I and *Sac*I. The repair template was created using oligos of FKS1rt_Fwd and S645P_Rev. Repair templates (10 μg) were purified and transformed along with linearized pV1200_*FKS1*sgRNA into *C. albicans* SC5314. The repair templates contained the desired point mutation (serine to proline at 645), and a silencing mutation at 635 (TTG, Leucine to TTA, Leucine) to prevent the Cas9 system repeatedly cutting the NGG site. A positive colony was selected by plating on an YPD agar 200 μg/ml nourseothricin, and then grown on YPD agar containing 0.5 μg/ml caspofungin. All positive transformations were confirmed by the DNA sequencing analysis as previously described (Lee et al., 2012). To create the dysfunctional *fks1* mutant, the repair template was produced by using oligos of FKS1rt_Fwd and *fks1*del_Rev. The repair template was purified and transformed along with linearized pV1200_*FKS1*sgRNA into *C. albicans* SC5314. Positive transformants were selected on an YPD with 200 μg/ml nourseothricin. All mutants were screened by Sanger sequencing with primers of Fks1_HS1_F and _R (Genewiz UK; https://www.genewiz.com/).

### Computational analysis of Fks1 or β-1,3-glucan synthase homologue proteins

The amino acid sequences of Fks1 or homologues in all reference strains was obtained from the *Candida* genome database (http://www.candidagenome.org), the *Saccharomyces* genome database (http://www.yeastgenome.org) or from the NCBI (http://www.ncbi.nlm.nih.gov) (Table 2). The multiple protein sequence alignment of homologue Fks1s in *Candida* species was performed by using the web-based analysis tool, MUSCLE (http://www.ebi.ac.uk/Tools/msa/muscle) using *S. cerevisiae* Fks1 as the comparator sequence. The phylogenetic tree of Fks1 was created after MUSCLE analysis, and then re-generated as an unrooted phylogenetic tree, using TreeDyn 198.3 (http://www.phylogeny.fr/). The transmembrane topology prediction of homologue Fks1 was performed via the web-tool, Phobius (http://phobius.sbc.su.se/index.html).

**Table 2:** Analysis of Fks1 β-1,3-glucan conserved central domain in *Candida* species.

Species	Strain name	Protein sequence info.	Protein length	Chromosome info.	Source	Amino acid position (putative β-1,3-glucan synthase components)	Domain facing
*C. albicans*	SC5314	C1_02420cp_a	1897	Chr1	CGD	701-1361	NON CYTOPLASMIC
*C. glabrata*	CBS 138	Cagl0k01034gp	1863	ChrG	CGD	685-1344	NON CYTOPLASMIC
*C. dubliniensis*	CD36	Cd36_02270p	1897	Chr1	CGD	701-1361	NON CYTOPLASMIC
*C. guilliermondii*	ATCC 6260	EDK37070	1882	Supercontig CH408155	Ensemble Fungi	713-1295	CYTOPLASMIC
*C. krusei*	ATCC 6258	AAY40291.2	1885	NA	NCBI	715-1368	NON CYTOPLASMIC
*C. orthopsilosis*	90-125	CCG22470	1902	Chr2	EnsembleFungi	726-1310	CYTOPLASMIC
*C. parapsilosis*	ATCC 22,019	Cpar2_106400p	1909	Chr1	CGD	733-1373	CYTOPLASMIC
*C. tropicalis*	MYA-3404	EER31878	1280	Supercontig3.6	EnsembleFungi	86-746	NON CYTOPLASMIC
*S. cerevisiae*	S288C	YLR342W	1876	Chr XII	SGD	720-1357	CYTOPLASMIC

CGD = *Candida* Geneome Database (http://www.candidagenome.org/).

EnsembleFungi = http://fungi.ensembl.org/index.html.

NCBI = National Centre for Biotechnology Information (http://www.ncbi.nlm.nih.gov/guide/).

SGD = Saccharomyces Genome Database (http://www.yeastgenome.org/).

Transmembrane domain analysis by using the web-based software, Phobius (http://phobius.sbc.su.se/).

## Declaration of Competing Interest

The authors declare that they have no known competing financial interests or personal relationships that could have appeared to influence the work reported in this paper.

## Figures and Tables

**Supplementary Fig. 3:**